# Case report: Immunotherapy in rare high TMB pancreatic acinar carcinoma

**DOI:** 10.3389/fonc.2024.1357233

**Published:** 2024-03-11

**Authors:** Guifu Wu, Yuting Fang, Deying Bi, Wenwei Yang, Yongkun Sun

**Affiliations:** ^1^Department of Medical Oncology, Beijing Chaoyang District Sanhuan Cancer Hospital, Beijing, China; ^2^National Cancer Center/National Clinical Research Center for Cancer/Cancer Hospital, Chinese Academy of Medical Sciences and Peking Union Medical College, Beijing, China

**Keywords:** PACC, immunotherapy, high TMB, adverse reaction, genetic testing

## Abstract

This case report details a patient with Pancreatic Acinar Cell Carcinoma (PACC), a rare malignancy with distinctive biological and imaging features. In the absence of standardized treatment protocols for PACC, we embarked on a diagnostic journey that led to the adoption of an innovative therapeutic regimen in our institution. A 45-year-old female patient presented with a pancreatic mass, which was histologically confirmed as PACC following a biopsy. Subsequent genomic profiling revealed a high tumor mutational burden (21.4/Mb), prompting the initiation of combined immunotherapy and targeted therapy. Notably, the patient experienced a unique adverse reaction to the immunotherapy—recurrent subcutaneous soft tissue nodules, particularly in the gluteal and lower limb regions, accompanied by pain, yet resolving spontaneously. Following six cycles of the dual therapy, radiological evaluations indicated a decrease in tumor size, leading to a successful surgical excision. Over a 20-month post-surgical follow-up, the patient showed no signs of disease recurrence. This narrative adds to the existing knowledge on PACC and highlights the potential efficacy of immunotherapy in managing this challenging condition, emphasizing the importance of close monitoring for any adverse reactions.

## 1 Introduction

Pancreatic acinar cell carcinoma (PACC) is a rare malignant exocrine tumor that originates from pancreatic acinar cells and terminal branches of the pancreatic duct, constituting only 1% of pancreatic neoplasms. It possesses distinctive biological behavior and imaging traits, including a large tumor size, a “pseudo-capsule”, central necrosis and hemorrhage, potent invasiveness, hypoxia, and vascular invasion (1, 2). Lymph node metastasis and distant metastasis frequently accompany it. The infrequency of PACC, compounded by the absence of prospective randomized clinical trials, has resulted in a lack of uniform treatment protocols among oncologists, although radical surgical resection with negative margins has shown a correlation with enhanced long-term survival (3).

Immunotherapy, a novel strategy that mobilizes the body’s immune defenses, has emerged as a pivotal treatment for certain intractable or metastatic cancers that do not respond to standard therapies. However, the efficacy of immunotherapy in treating PACC remains uncertain. In this report, we detail a case of PACC characterized by a high Tumor Mutational Burden (TMB), initially classified as inoperable. Subsequent to a regimen combining immunotherapy and targeted treatment, the patient underwent a successful R0 resection, leading to an extended recurrence-free survival. Another point worth mentioning, a rare adverse reaction occurred during immunotherapy: recurring systemic subcutaneous soft tissue nodules (primarily in the buttocks and lower limbs, presenting with pain and resolving spontaneously).

## 2 Case presentation

**Figure 1:**
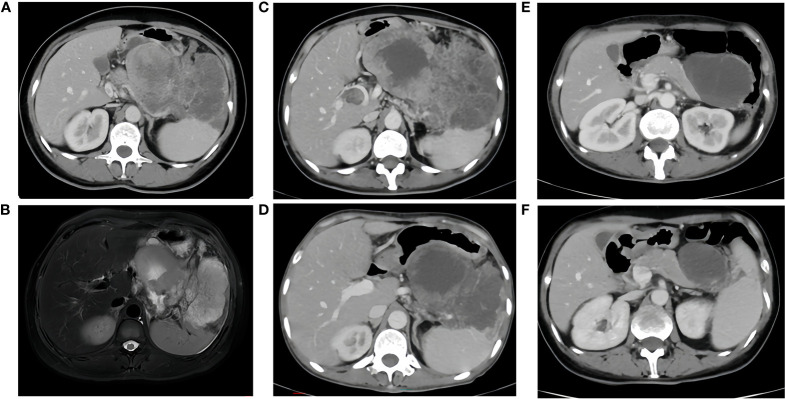
**(A, B)**, Sep. 17, 2020 CT/MR A large irregular mass fused between the gastric cavity, pancreas and spleen, with the largest cross-section measuring approximately 13x16x8cm, it was considered malignant and invaded the splenic hilum, pancreas and local gastric wall. There were intravascular tumor thrombi in the splenic vein and portal vein, multiple nodules in the abdominal cavity, mesentery and greater omentum, suggesting metastasis. **(C)**, before immunotherapy, size of the tumor: 17.8x10cm. **(D)**, after two cycles of immunotherapy, size of the tumor: 11.0x8.2cm. **(E)**, after four cycles of immunotherapy, size of the tumor: 8.0x6.2cm. **(F)**, after six cycles of immunotherapy, size of the tumor: 6.2x4.4cm.

**Figure 2:**
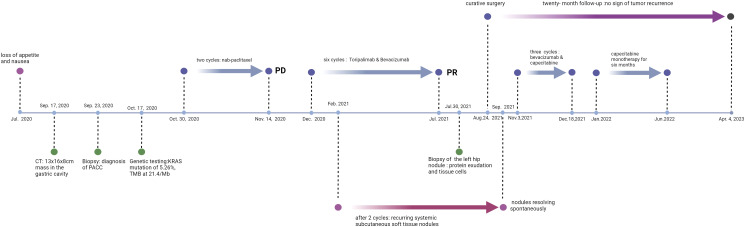
Timeline.

A 45-year-old female patient presented at our hospital, complaining of persistent loss of appetite and nausea for over a month, worsening over the past two weeks. Her family, drug, past medical, and past surgical history were negative. An ultrasound examination had identified a large abdominal mass (Sep., 2020). A subsequent CT/MR scan (Sep. 17, 2020) disclosed a large irregular mass fused among the gastric cavity, pancreas, and spleen, with its largest cross-section measuring approximately 13x16x8cm (**Figure 1**). An ultrasound-guided biopsy (Sep. 23, 2020) of the tumors in the left upper abdomen displayed epithelial-derived tumors with glandular tubule-like and papillary structures. Immunohistochemistry results, including AE1/AE3 (3+), CK7(2+), ACT (3+), AAT (3+), CK20(-), CD56(-), Syno (-), chrA (-), CD10(-), B-Catenin (+), ER (-), PR (1+), WT-1(-), CATA3(-), CD34(-), and Ki-67 (+35%), led to the diagnosis of PACC. From October 30, 2020, to November 14, 2020, the patient completed two cycles of chemotherapy with “nab-paclitaxel 200 mg d1/14 days repeat” (**Figure 2**). Despite tolerating this regimen, a subsequent CT scan indicated an increase in the size of the mass, with its largest cross-section measuring approximately 17.8x10 cm, classified as progressive disease (PD).

**Figure 3:**
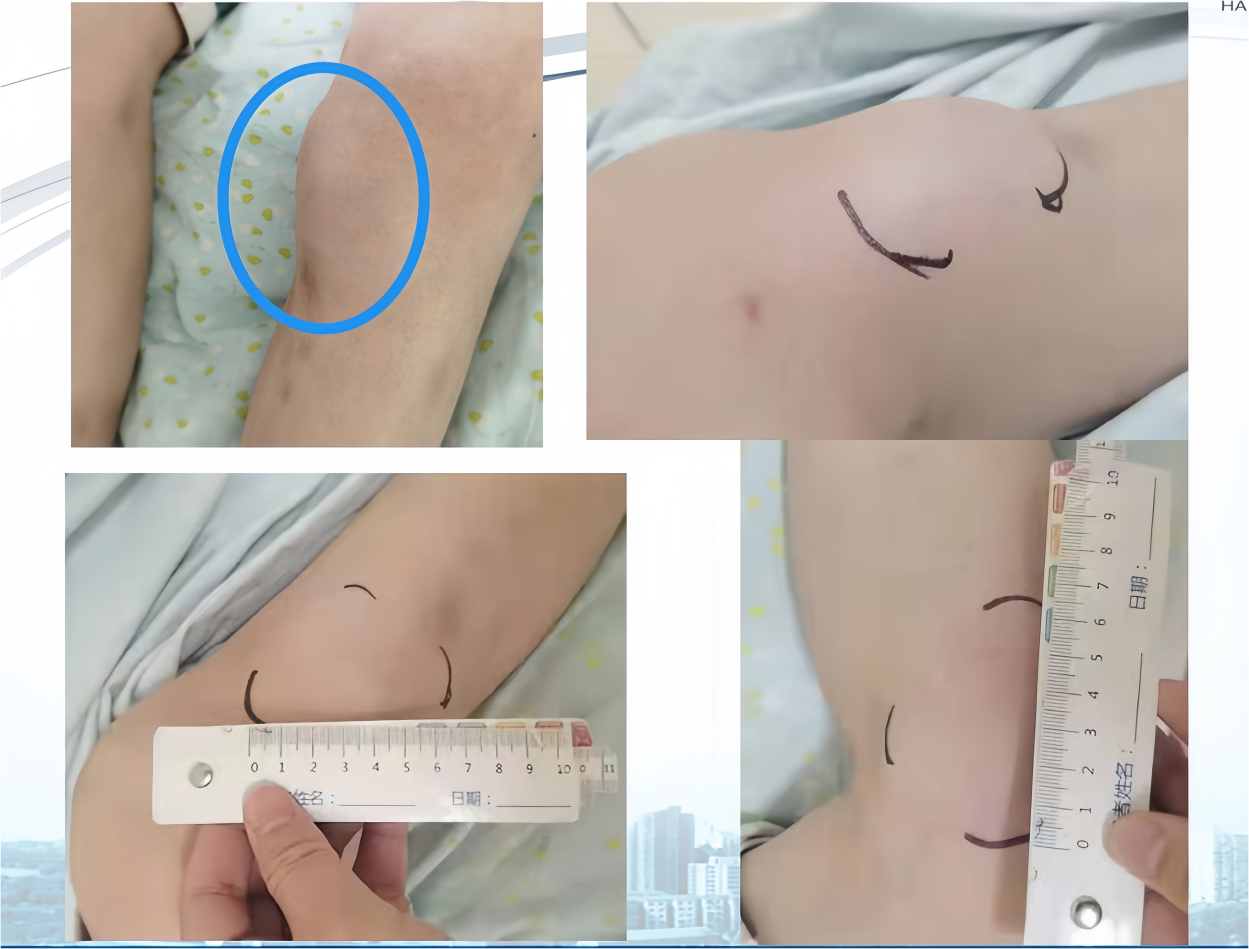
(Left Lower Limb) Systemic subcutaneous soft tissue nodules post-immunotherapy (primarily in the buttocks and lower limbs).

**Figure 4:**
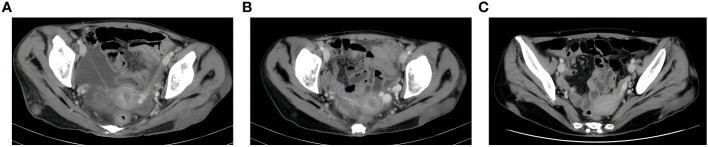
CT Soft tissue nodule of left buttock. **(A)**, after two cycles of immunotherapy, size of soft tissue nodules: 1.9x6.3cm. **(B)**, after four cycles of immunotherapy, size of soft tissue nodules:1.5x6.8cm. **(C)**, after six cycles of immunotherapy, size of soft tissue nodules:1.6x5.8cm.

Next-generation sequencing results revealed a KRAS mutation with a mutation frequency of 5.26%; HRD positive, 50 points, TMB at 21.4/Mb, indicating high TMB. PD-L1–positive expression and high TMB have been reported to potentially predict improved response of tumors to immunotherapy (4). Despite the absence of PD-L1 expression data in this patient’s biopsy pathology, the high TMB suggests a possible responsiveness to immunotherapy. Lacking standard treatment and with request to try immunotherapy by the patient, we utilized Toripalimab, an anti-PD-1 inhibitor, with the consideration of medication availability and the patient’s financial capacity. From December 05, 2020, to July, 2021, the patient was administered six cycles of “Toripalimab 240mg d1 + Bevacizumab 300mg d1/21 days repeat”. The tumor response was assessed by CT scans every two cycles. The tumor size decreased progressively and was reduced to 6.2x4.4 cm after six cycles, resulting in a partial response (PR) (**Figure 1**). Notably, following two cycles of immunotherapy, the patient experienced recurring systemic subcutaneous soft tissue nodules, primarily in the buttocks and lower limbs, which presented with pain and resolved spontaneously (**Figures 3** and **4**). A guided ultrasound of the left hip nodule showed protein exudation and tissue cells but did not support metastasis. Based on the immunotherapy application, we suspect that the nodules might be an adverse reaction caused by the immunotherapy.

Following an MDT discussion, surgical intervention was recommended. With the patient’s consent, she underwent a surgery “splenectomy with distal pancreatectomy + partial colectomy + partial gastrectomy” on Aug. 24, 2021. The resected tissue was submitted for pathological analysis, which confirmed a diagnosis of PACC. Notably, the tumor exhibited extensive degeneration and necrosis, in conjunction with cystic transformation, interstitial fibrosis, and infiltration by inflammatory cells—a constellation of findings indicative of post-therapeutic alterations. The greatest dimension of the remaining tumor reached 2.5 cm. It was found to be adherent to both the gastric and intestinal walls but refrained from direct infiltration into their tissue. The tumor extended to the spleen’s capsule, yet spared the splenic parenchyma, with no evidence of invasion into the vascular or neural structures. The pancreatic, gastric, and intestinal margins were free of cancer, with no metastatic cancer found in the lymph nodes (0/17); ypT2N0. The treatment elicited a notable response, however, it fell short of achieving a complete pathologic response.

The resected tissue was further submitted for immunohistochemical and genetic testing. The immunohistochemistry results were as follows: MLH1 (+), PMS2 (+), MSH2 (+), MSH6(+), P53 (+20%), PD-L1 Neg (-), PD-L1 (22C3) (CPS=0), AAT (3+), ACT (3+), CK7 (3+), CK19(-), AE1/AE3 (3+). Genetic testing yielded the following results: an APC gene exon 16 mutation; KRAS, NRAS, PIK3CA, and BRAF were all negative; no ALK, FGFR, ROS1, RET, NTRK gene translocations were shown; no HER2, CMET, EGFR gene mutations were shown; TMB was 1 mutation/Mb; MSS type.

After achieving R0 resection, we anticipated that the continuation of immunotherapy would not confer additional benefits, but rather increase the patient’s economic burden and adverse reactions. Therefore, we opted not to proceed with immunotherapy post-surgery, but used chemotherapy and targeted therapy instead. The patient received three cycles of “bevacizumab 400mg on day 1 + oral capecitabine 1g in the morning and 1.5g in the evening, repeated on days 1-14 every 21 days” from Nov. 3, 2021, to Dec. 18, 2021. “Oral capecitabine 1g in the morning and 1.5g in the evening, repeated on days 1-14 every 21 days” monotherapy was continued for six months, from January to June, 2022. A Grade II gastrointestinal reaction was observed. Regular follow-ups were conducted, and no signs of tumor recurrence were found during the 20-month follow-up period after surgery (last examination date: Apr. 4, 2023).

## 3 Discussion

### 3.1 Analysis of the reasons for the effectiveness of immunotherapy in this case

Immunotherapy has emerged as a critical component of cancer treatment, rapidly gaining traction and widespread use across a range of solid tumors. The efficacy of immunotherapy as a standalone treatment is, however, rather limited. In clinical practice, it is often used synergistically with chemotherapy, targeted therapy, and other treatments to amplify the benefits for patients. Numerous studies have established a strong correlation between the effectiveness of immunotherapy and genetic factors. PD-L1, a protein found on cancer and immune cells, interacts with PD-1 receptors to regulate immune responses. It is observed that a higher expression of PD-L1 often correlates with more favorable outcomes in immunotherapy (5). Tumor Mutational Burden (TMB), which refers to the number of mutations within a tumor’s DNA, has similarly been linked to better responses to immunotherapy, as tumors with high TMB may present more antigens to the immune system (5). Microsatellite instability (MSI) or DNA mismatch repair deficiency (dMMR) is another critical biological marker, significantly predicting the effectiveness of immunotherapy in patients with solid tumors. This condition results from the accumulation of mutations due to the failure of the DNA repair system, making tumors with MSI/dMMR more susceptible to immune checkpoint inhibitors (6). Furthermore, alterations in the POLE/POLD1 genes, which are involved in DNA proofreading and repair, have also been associated with favorable responses to PD-1 inhibitors (7).

Immune checkpoint molecules function to inhibit T-cell cytotoxicity against tumor cells, thereby restraining the antitumor immune response driven by neoantigens. Immune checkpoint inhibitors (ICIs), including PD-1, PD-L1, and CTLA-4 antibodies, target specific immune checkpoint molecules to restore T-cell cytotoxicity against tumor cells, thus initiating an antitumor immune response (8). Tumors characterized by high TMB typically present elevated levels of neoantigens. These neoantigens, arising from tumor-specific somatic mutations and their degradation products, are displayed on the tumor cell surface via major histocompatibility complexes. This presentation is pivotal in activating T cells, thereby potentially enhancing the efficacy of ICIs in targeting these cancer cells (5). Since, 2015, extensive clinical research has delved into the predictive significance of Tumor Mutational Burden (TMB) in the efficacy of immunotherapy for solid tumors. A pivotal study in this field, KEYNOTE-158, encompassed 1,032 patients with refractory solid tumors spanning 10 different cancer types. This study revealed a notable overall objective response rate (ORR) of 29% to Pembrolizumab, a PD-1 antibody, in patients with high TMB, compared to a mere 6% ORR in those with low TMB (9). This finding led to the FDA approval of Pembrolizumab for patients with unresectable or metastatic solid tumors exhibiting high TMB and disease progression post-prior treatment, categorizing high TMB as tissue TMB ≥ 10 mutations/Mb. High TMB has been correlated with enhanced progression-free survival (PFS) in cancer patients treated with ICIs. As research into TMB advances, it is increasingly being recognized as a potential predictive marker for ICI efficacy, thereby aiding in the optimization of treatment strategies for cancer patients undergoing immunotherapy (5). In this case, the genetic test showed a TMB of 21.4/Mb, qualifying as high TMB, which may have contributed to the benefit gained from immunotherapy. The significant response achieved after combined immunotherapy and targeted therapy facilitated a successful transition to surgical intervention. However, the potential of TMB as a predictive indicator of therapeutic efficacy for PACC still requires confirmation through additional clinical data. Although this case illustrates the benefits of combining immunotherapy with targeted therapy, whether immunotherapy can become a treatment standard for PACC needs further investigation and validation.

### 3.2 Is the recurrent multiple subcutaneous nodules related to immunotherapy?

Following immunotherapy, the patient in this report experienced recurrent subcutaneous soft tissue nodules and widespread joint pain. According to existing data, PACC can occasionally present with lipase hypersecretion syndrome, with an incidence rate of 10%-15%. Excessive tumor-derived lipase secretion enters the bloodstream, leading to manifestations such as elevated serum lipase levels, subcutaneous fat necrosis, polyarthralgia, and increased peripheral blood eosinophils. Subcutaneous fat necrosis is the most distinctive symptom. The exact pathogenesis is not fully understood, but one potential mechanism involves excessive production of pancreatic protease and lipase altering vascular permeability. Lipase hydrolyzes lipids in the cell membrane and cytoplasm of fat cells, inducing fat necrosis and nodule formation (10).

Adverse reactions to immunosuppressants commonly impact the digestive, endocrine, and nervous systems, yet the involvement of subcutaneous soft tissues is relatively rare. In this case report, the patient developed systemic subcutaneous nodules following immunotherapy, which self-resolved and typically dissipated before the subsequent immunotherapy cycle. The biopsy pathology excluded tumor metastasis. While clinical considerations suggest these could be immune reactions, the underlying mechanisms are not yet fully understood. Determining whether these phenomena are linked to abnormal fat metabolism or other etiologies necessitates further investigation.

## 4 Summary

PACC is a rare malignancy, resulting in limited data to guide treatment strategies. Clinically, surgical resection is often recommended for PACC patients. Surgical resection with negative margins has been associated with improved long-term survival (3). However, the lack of prospective randomized controlled trial (RCT) evidence, established guidelines, and consensus on management poses challenges in standardizing treatment for non-resectable cases. For early-stage PACC, radical resection is advised. The feasibility of radical surgery is determined by factors such as tumor location, size, extent, lymph node and distant metastasis status, the patient’s surgical tolerance, and patients’ willingness (3).

Combining surgery with chemotherapy and radiation is a prevalent clinical approach, although prospective data to guide chemotherapy protocols are still absent. For metastatic PACC, chemotherapy regimens used for PDAC have been employed, incorporating gemcitabine, 5-FU, oxaliplatin, CPT-11, and S-1, or combinations thereof (11). Currently, the development of targeted therapy for PACC is slow. While therapies targeting BRAF, BRCA2 gene mutations, or MMR defects may benefit PACC patients, further investigation is required. Immunotherapy is broadly used in solid tumors. More clinical data is needed to confirm whether high PD-L1 expression and high TMB can serve as predictive markers for the efficacy of immunotherapy in PACC. The case reported here benefited from a combination of immunotherapy and targeted therapy. However, whether immunotherapy can become a standard treatment for PACC requires further research and verification.

## Data availability statement

The original contributions presented in the study are included in the article/supplementary material. Further inquiries can be directed to the corresponding author.

## Ethics statement

Written informed consent was obtained from the individual(s) for the publication of any potentially identifiable images or data included in this article.

## Author contributions

GW: Writing – original draft, Methodology, Data curation. YF: Writing – review & editing, Writing – original draft, Software, Investigation, Conceptualization. DB: Writing – review & editing, Resources, Data curation. WY: Writing – review & editing, Resources, Investigation, Data curation. YS: Writing – review & editing, Validation, Supervision, Resources, Methodology, Investigation, Funding acquisition, Conceptualization.
